# Supplementary material for: Impact ionization by hot carriers in a black phosphorus field effect transistor
Source: Nat Commun. 2018 Aug 24;9:3414. doi: 10.1038/s41467-018-05981-0 (PMC6109174; doi:10.1038/s41467-018-05981-0)
Supplement: Supplementary file 1 — Supplementary Information [file 41467_2018_5981_MOESM1_ESM.pdf]

Supplementary Information

Impact ionization by hot carriers in black phosphorus field effect transistor

Faisal Ahmed[1,2], Young Duck Kim[3,4], Yang Zheng[2], Pan He[5], Euyheon Hwang[2], Hyunsoo Yang[5], James Hone[3], and Won Jong Yoo[1,2,*]

[1]School of Mechanical Engineering, Sungkyunkwan University, 2066, Seobu-ro, Jangan-gu, Suwon, Gyeonggi-do, 16419, Korea.

[2]Department of Nano Science and Technology, SKKU Advanced Institute of Nano-Technology (SAINT), Sungkyunkwan University, 2066, Seobu-ro, Jangan-gu, Suwon, Gyeonggi-do, 16419, Korea.

[3]Department of Mechanical Engineering, Columbia University, New York, New York 10027, USA.

[4]Department of Physics, Kyung Hee University, Seoul 02447, Korea.

[5]Department of Electrical and Computer Engineering, and NUSNNI, National University of Singapore, Singapore 117576, Singapore.

*Corresponding email: yoowj@skku.edu

Supplementary Figures

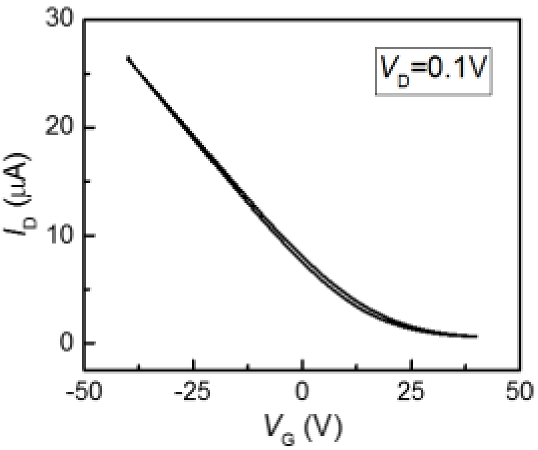

Supplementary Figure 1 **Hysteresis of hBN encapsulated BP device**. The measured hBN encapsulated BP devices exhibited almost negligible hysteresis at $V_D = 0.1V$ thanks to ultra-clean and atomically flat surface of hBN, as it discouraged charge traps along the BP-hBN interface[1].

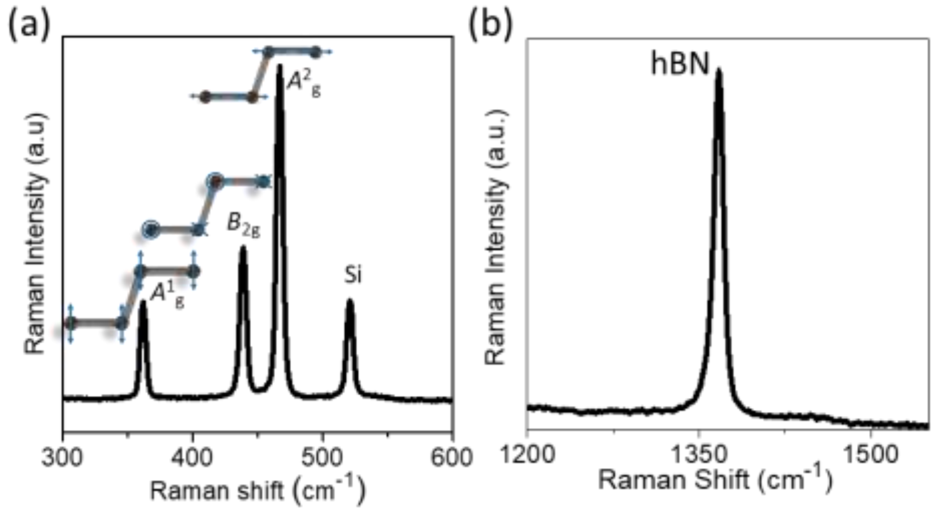

Supplementary Figure 2 **Raman spectra of BP and hBN. (a)** Raman signals obtained from a multilayer BP flake, together with the atomic vibrations along their respective directions. **(b)** Raman peak collected from multilayer hBN flake.

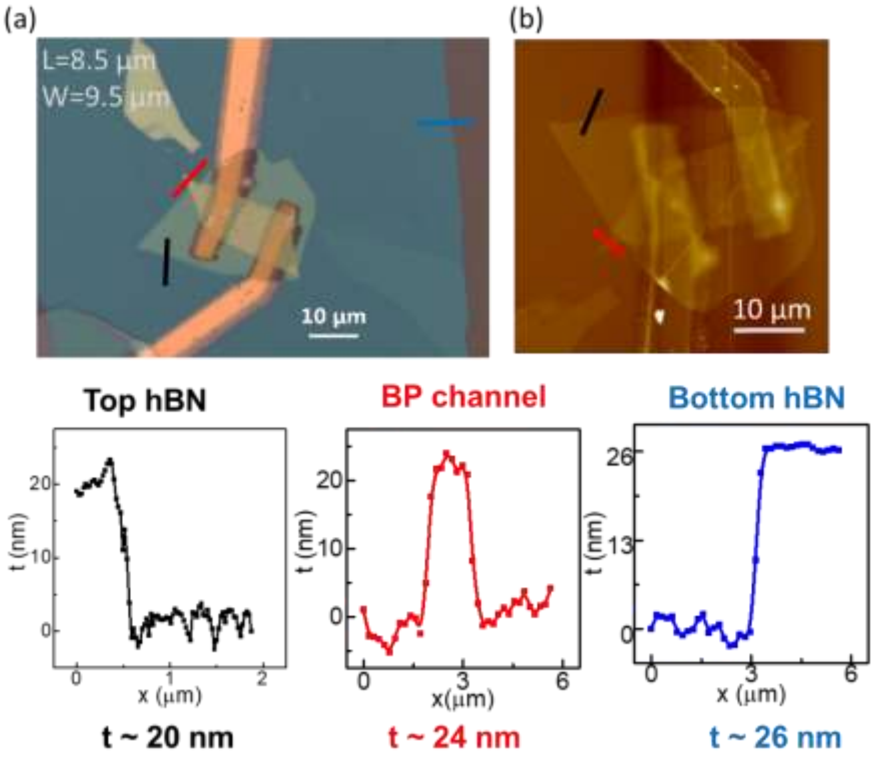

Supplementary Figure 3 **Thickness information of devices. (a)** and **(b)** are OM and AFM images of hBN encapsulated BP device, where black, red and blue lines and plots denotes thicknesses of top hBN, BP and bottom hBN respectively.

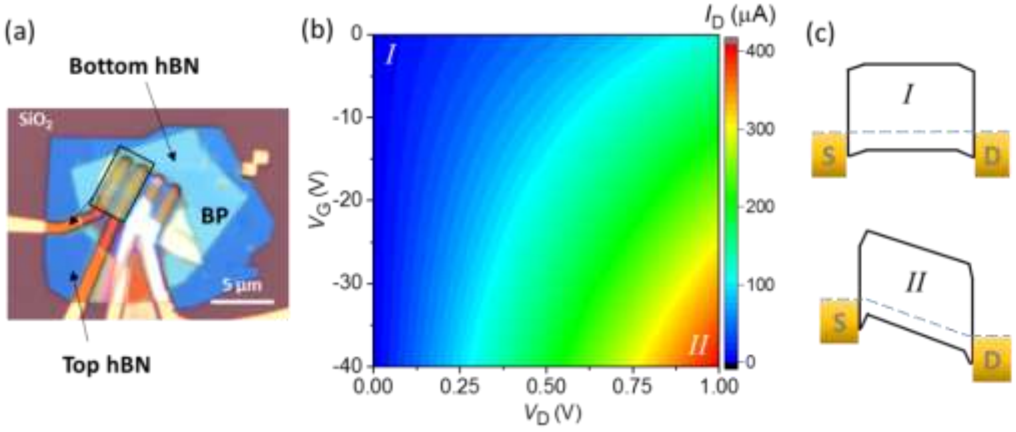

Supplementary Figure 4 **Low electric field results of device (2). (a)** OM image of hBN encapsulated BP device 2, where the black rectangle indicates measured device. **(b)** Low electrical field 2D contour plot of BP device. **(c)** Band position of BP at specific bias condition.

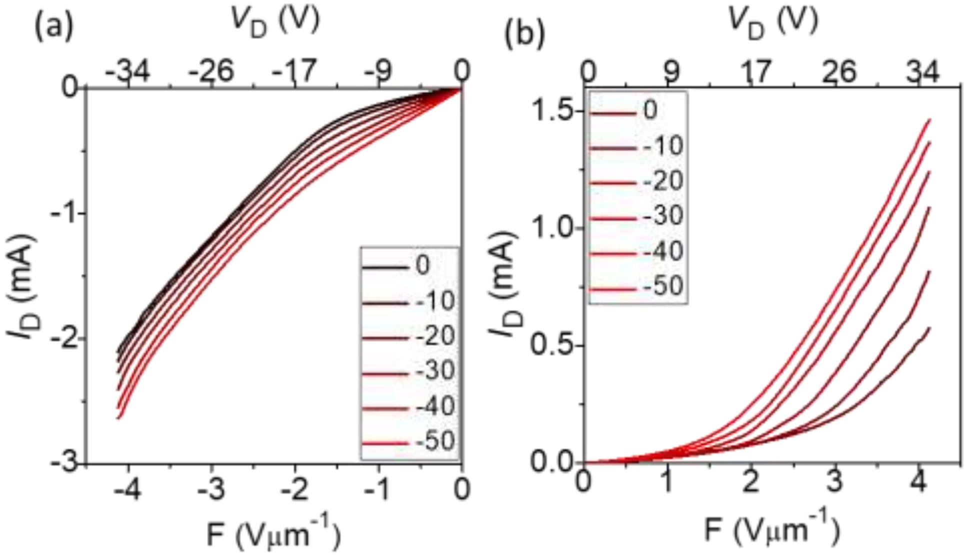

Supplementary Figure 5 **Gate dependent high electrical field behavior. (a)** and **(b)** are high field output plots for positive and negative V_D regions respectively at given V_G.

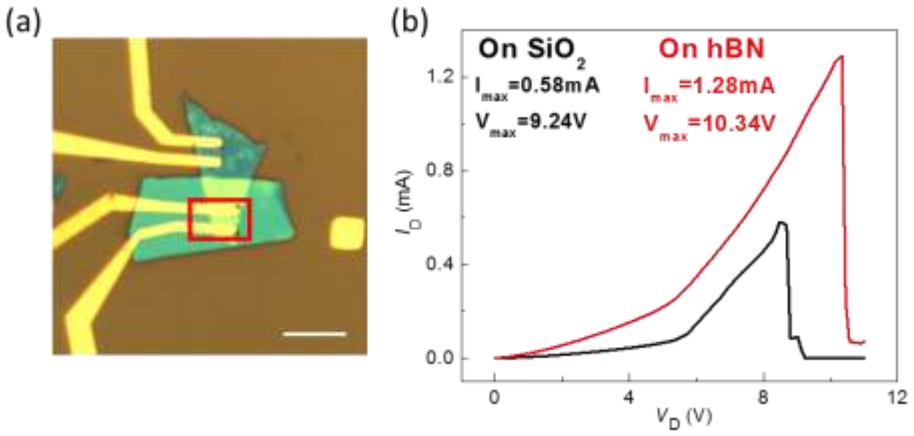

Supplementary Figure 6 **Effect of substrate to *I-V* behavior. (a)** OM image of BP devices on SiO_2 and hBN/SiO_2 substrates, with scale bar of 5 nm. **(b)** Electrical breakdown of BP devices on SiO_2 and hBN/SiO_2 substrates showing absence of current saturation.

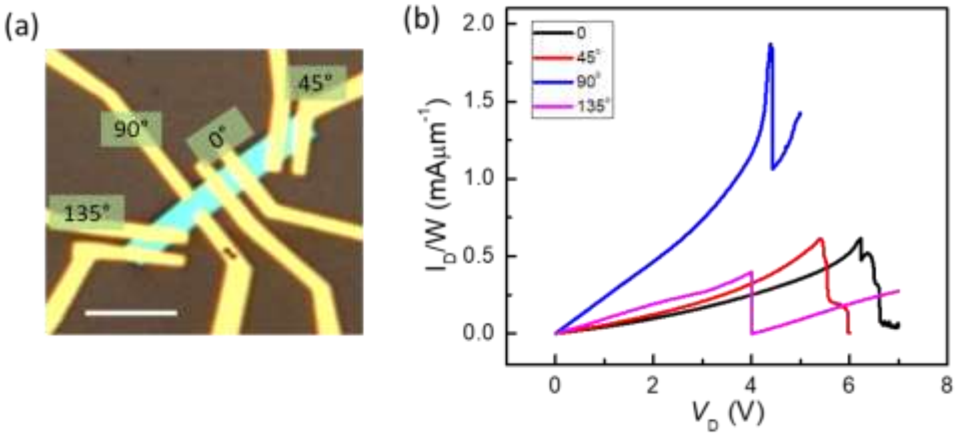

Supplementary Figure 7 **Effect of anisotropy to *I-V* behavior. (a)** OM image of BP devices along different in-plane directions. Scale bar is 10 μm. **(b)** The *I-V* plots of BP devices at given angles up to breakdown.

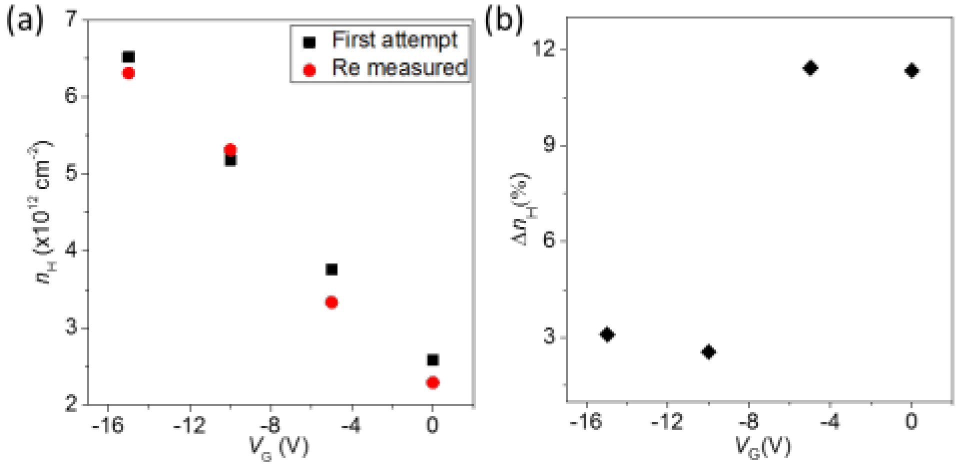

Supplementary Figure 8 **Effect of Joule heating to carrier density. (a)** Hall carrier density measured initially and after the effect of Joule heating at I_D = 0.1 mA. **(b)** Change in carrier density at different V_G.

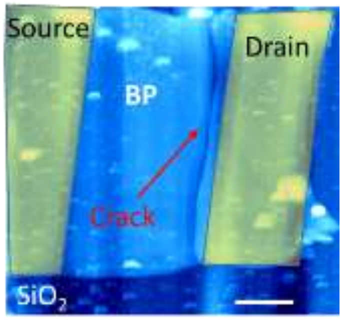

Supplementary Figure 9 The false color AFM image of broken BP device on SiO_2 substrate. Scale bar is 2 µm. The broken device shows a crack along the drain contact.

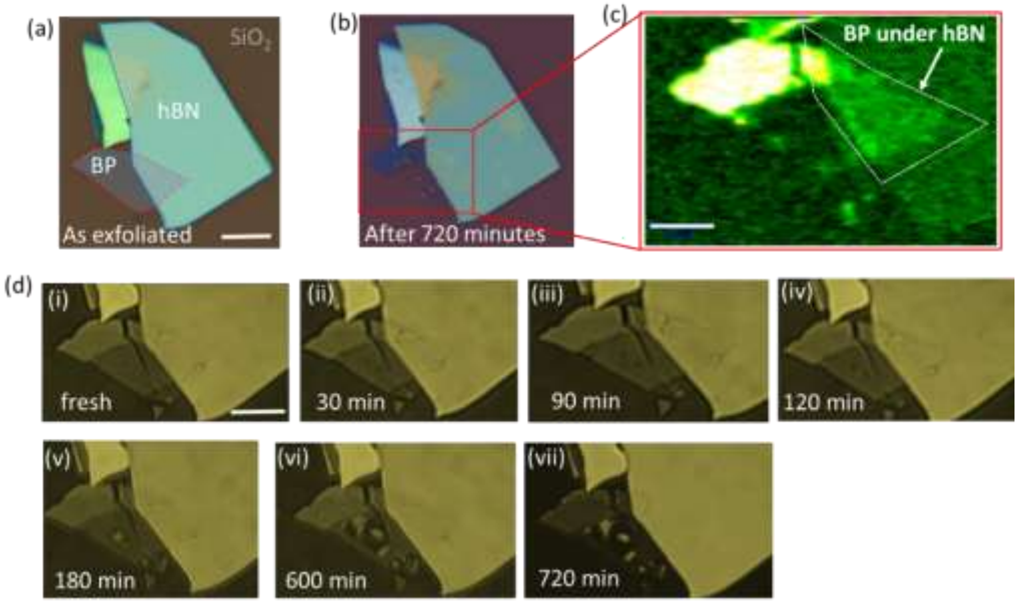

Supplementary Figure 10 **Optical microscope (OM) images of bare and hBN passivated BP flakes with time. (a)** OM image of targeted flakes soon after exfoliation and transfer in glove box where red shaded boundary region denotes few layers BP flake partially covered with hBN, and blue shaded region is hBN. Scale bar is 5 µm. **(b)** OM image of same flakes after 720 minutes exposure to ambient conditions. The exposed BP flake disappeared in that span of time. **(c)** Spatial Raman intensity map (A^2_g mode of BP) of red rectangle-indicated region shown in (b), confirming existence of BP under hBN, here scale bar is 3 µm. **(d)** False color OM images taken after given time span from (i) - (vii).

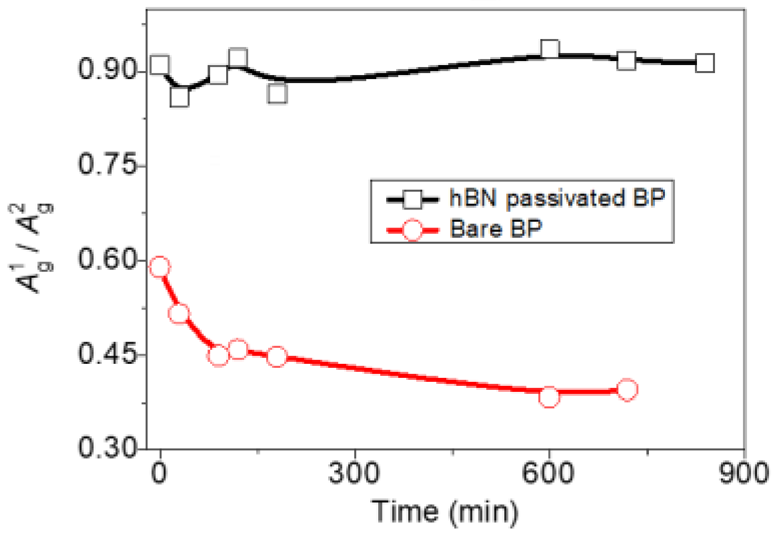

Supplementary Figure 11 **Time dependent Raman spectra of BP.** Raman intensity peak ratio ($A^1{}_g/A^2{}_g$) as a function of time collected from bare and hBN passivated BP flake.

Supplementary Tables

Supplementary Table 1. Reported current saturation in BP devices

#	Supplementary References number	BP channel		Gate dielectric		
		Gate length (µm)	Thickness (nm)	Material	Dielectric constant	Thickness (nm)
1	[2] L. Li et al., *Nat. Nanotechnol.* 2014, *9*, 372–377	4.5	5	SiO_2	3.9	90
2	[3] W. Zhu et al., *Nano Lett.* 2015, *15*, 1883–1890	2.7	15	Al_2O_3	10	25
3	[4] H. Wang et al., *Nano Lett* 2014, *14*, 6424–6429	0.3	8.5	HfO_2	16.5	21
4	[5] S. Das et al., *ACS Nano* 2014, 8, 11730–11738	2	1.9	SiO_2	3.9	20
5	[6] W. Zhu et al., *Nano Lett.* 2016, *16*, 2301–2306	0.5	13	Al_2O_3	10	25

Supplementary Table 2. Thickness information of BP measured devices

Device #	Geometry	Thickness (nm)			Remarks
		Bottom hBN	BP	Top hBN	
1	hBN/BP/hBN	26	24	20	Two-probe device (1); Data set shown in Figs. 1b and 1c for negative V_D breakdown
2	hBN/BP/hBN	30	36	25	Two-probe device (2); Data set shown in Fig. 1d in main text and Supplementary Figure 4 for positive V_D breakdown.
3	hBN/BP/hBN	15	28	9	Hall bar device; Data set shown in Figs. 3c and 3d
4	hBN/BP/hBN	40	30	18	Hall bar device; Data set shown in Figs. 4a and 4b
5	hBN/BP	25	6	-	Two terminal devices; Data set shown in Supplementary Figure 6 for different dielectric comparison.
6	SiO_2/BP	285	33	-	Two terminal devices; Data set shown in Supplementary Figure 7 for anisotropic breakdown comparison.

Supplementary Note 1

Current saturation in BP devices

Previously there have been number of publications, wherein, the observation of current saturation in few layer BP devices is reported. In most of these studies, a very thin and/or high-*k* gate dielectric material such as Al_2O_3 and HfO_2 and narrow BP channel are used to realize very strong dielectric charge screening effect as summarized in Supplementary Table 1. These kinds of structures are helpful for radio-frequency applications but at the same time causes fabrication complexities and gate leakage problems.

In this study, we aim to study the high field intrinsic carrier transport in BP devices. For this, we encapsulated BP flakes with hBN to minimize the external effects. hBN is an ultra-clean and atomically smooth dielectric thus enables sharp interface. In addition to this, both hBN and SiO_2 are low dielectric constant materials (3.4 and 3.9 respectively), that induce weak dielectric charge screening to the BP channel. That is one of the factors that we observe super-linear *I-V* characteristics instead of current saturation in hBN encapsulated and SiO_2 supported BP devices studied here.

Supplementary Note 2

Raman spectra of BP and hBN

We acquired Raman spectra of multilayer BP and hBN flakes supported on SiO_2 substrate. For BP flake, we observed four prominent Raman peaks, three for BP and one for Si, as shown in Supplementary Figure 2a. Of the three Raman modes of BP, the A^2_g and B_{2g} modes at 362 cm^{-1} and 439 cm^{-1}, respectively, corresponded to the atomic vibration of phosphorus atoms along their corresponding in-plane directions, *i.e.*, the zigzag and armchair. The third Raman mode, A^1_g, corresponded to out-of-plane vibration and appeared at 467 cm^{-1} (supplementary reference 7). Besides this, we also compiled Raman spectra of one of the

exfoliated multilayer hBN flake, as shown in Supplementary Figure 2b. hBN exhibits only one characteristics Raman peak at 1366 cm^{-1} corresponding to E_{2g} vibration mode that is analogous to the G mode in graphene. However, the absence of D peak corresponds to lack of Kohn anomaly in hBN[8].

Supplementary Note 3

Thickness information of BP devices

Firstly, the candidate flakes of BP and hBN were selected by color contrast and their detailed thickness characterization was carried out by atomic force microscopy (AFM). The thickness information of the representative BP devices is compiled in Supplementary Table 2. As an example, the optical microscopic (OM) and AFM images of two terminal hBN encapsulated BP device 1 are shown in Supplementary Figures 3a and 3b. This device was measured to acquire the data set shown in Figs. 1b and 1c of main text. The height profile of individual flake scanned by AFM is shown in the plots, where, hBN top, BP channel and hBN bottom are 20 nm, 24 nm and 26 nm thick respectively, as indicated by black, red and blue colors respectively.

Supplementary Note 4

Low field characterization of device (2)

The high electrical field results measured towards the positive V_D range, shown in Fig. 1d in the main text, were obtained from multilayer hBN encapsulated 36 nm thick BP device (2) as shown in Supplementary Figure 4. The low field 2D contour plot depicted hole dominated transport as normally observed in thicker BP flakes. The carrier transport can be further explained by energy band diagram. When V_G is swept from 0 to –40 V, the bends were bent upwards inducing smaller and narrower barrier for hole that will increase current level of the BP device.

Afterwards, we increased the applied electrical field to very high positive V_D region, as shown in Fig. 1d of main text. Where, the current level kept rising with the applied electrical field until Joule breakdown occurred. That confirmed the observation of super-linear *I-V* behavior and absence of current saturation along positive V_D sweep.

Supplementary Note 5

Gate dependent high field transport

The carrier density is one of the critical parameters for CM phenomenon. CM is usually preferable at low carrier density, since it results in strong electron-electron interactions. To study the possible effect of carrier density to super-linear *I-V*, we applied different gating conditions (0 to –50 $V_{G,}$) to modulate the electrostatic doping concentration in BP channel at very large field in both forward and reverse bias directions, as shown in Supplementary Figures 5a and 5b respectively. For all the applied biasing conditions, our measured BP device showed super-linear behavior. However, the difference in the obtained current levels towards positive and negative V_D regions is probably due to different nature of band alignments in p-dominated BP channel[5]. In addition to this, we computed gate induced carrier density, using Supplementary equation (1),

$$n_G = C \times |V_G - V_{TH}|, \quad (1)$$

where V_{TH} is threshold voltage, C is the oxide capacitance, that is 1.05×10^{-8} Fcm^{-2} obtained by assuming a parallel-plate capacitor model by considering hBN and SiO_2 dielectrics. The maximum n_G value is 4×10^{12} cm^{-2} at $V_G = -50$V, that is still a small carrier concentration value induced by the gate dielectrics in BP channel. It is highly important to note here that the Hall carrier density (n_H) at very high electrical field in Fig. 3d is several times higher than gate induced carriers in BP channel. It is, therefore, confirmed that the additional charge carriers inside the BP channel are due to the CM by impact ionization by hot carriers.

Supplementary Note 6

Effect of substrate to super-linear *I-V* behavior

In order to confirm the possible effect of substrate to the super-linear behavior, we fabricated two devices on 6 nm thick BP flake, one directly on 285 nm SiO_2 substrate, and the other on 25 nm hBN supported flake, as shown in Supplementary Figure 6. The BP devices showed increasing *I-V* behavior instead of current saturation at high field regardless of nature of substrate used. However, hBN supported BP devices showed higher breakdown current density and field endurance due to structurally and thermally intimate BP/hBN interface[9].

Moreover, the devices shown below are supported on two different substrates which induce different screening effect. Here, both the dielectric materials used (hBN and SiO_2) are low-*k* dielectric materials, therefore, they will exhibit weak charge screening effect to BP channel.

Supplementary Note 7

Anisotropy dependent super-linear behavior of BP

Due to puckered structure of BP, it induces anisotropy along its in plane, resulting in different properties along its in-plane directions. BP shows different optical, physical, electronic and thermal properties along armchair and zigzag directions[10]. By keeping in mind this fact, we prepared several devices along different crystalline directions as shown in Supplementary Figure 7 to check the possible effect of orientation to current saturation. The high field transport results indicate the appreciable anisotropy in electrical conductivity, but still no sign of current saturation was realized. This concludes two main facts about BP; i) Super-linear behavior is independent of crystalline orientation, ii) the anisotropy of BP is intact even at large electric field.

Supplementary Note 8

Effect of Joule heating on carrier density

We observed a little change in carrier density after Joule heating effect as shown in Supplementary Figure 8. Our results showed approximately 10 ~ 12% deviation especially for V_G greater than – 6V, perhaps due to small carrier density and slightly higher R_c at high V_G. Nonetheless, 10 ~ 12% change is very small as compared to increase in carrier density at high field as shown in Fig. 3d in main manuscript. The increase in current level and improvement in R_c was previously realized for graphene devices mainly due to current induced annealing that leads to cleaning of interface[11]. However, the slight change in carrier density realized in our BP devices suggests a little effect of Joule heating towards interface cleaning and contact improvement.

Supplementary Note 9

Aging test of BP flakes

BP is notorious for its environmental sensitivity, and therefore, we performed aging test to monitor the degradation of BP and the effectiveness of hBN capping. We exfoliated few layer BP flake (7 ~ 8 nm) on SiO_2, and partially covered it with 25 nm thick hBN flake inside the glove box, as shown in Supplementary Figure 10a. Surprisingly, the naked BP flake degraded so fast that it completely disappeared just after twelve hours of high humid (75%) ambient exposure, as shown in Supplementary Figure 10b. This is further confirmed by spatial Raman intensity mapping (A^2_g mode of BP) that witnesses the vanishing of naked BP flake, while BP flake still exists underneath hBN, as shown in Supplementary Figure 10c. For more details, see temporal sequence of optical microscope images in Supplementary Figure 10d.

Raman spectroscopy is a non-invasive and highly effective technique to characterize the material qulaity[12]. Therefore, we acquired time dependent Raman characteristics of exposed

and hBN passivated BP flake to monitor its degradation in the ambient conditions. From our obtained results, we plotted the ratio of deconvoluted $A^1{}_g$ and $A^2{}_g$ modes of BP against time, see Supplementary Figure 11. Interestingly, a decreasing trend is observed for the bare BP flake, that is the signature of oxidations. However, the passivated BP flake shows stubborn spectra, thanks to effective passivation of BP by hBN.

Supplementary References

1. Dean, C. R., Young, A. F., Meric, I., Lee, C., Wang, L., Sorgenfrei, S., Watanabe, K., Taniguchi, T., Kim, P., Shepard, K. L. & Hone, J. Boron nitride substrates for high-quality graphene electronics. *Nat. Nanotechnol.* **5,** 722–726 (2010).
2. Li, L., Yu, Y., Ye, G. J., Ge, Q., Ou, X., Wu, H., Feng, D., Chen, X. H. & Zhang, Y. Black phosphorus field-effect transistors. *Nat. Nanotechnol.* **9,** 372–377 (2014).
3. Zhu, W., Yogeesh, M. N., Yang, S., Aldave, S. H., Kim, J. S., Sonde, S., Tao, L., Lu, N. & Akinwande, D. Flexible black phosphorus ambipolar transistors, circuits and AM demodulator. *Nano Lett.* **15,** 1883–1890 (2015).
4. Wang, H., Wang, X., Xia, F., Wang, L., Jiang, H., Xia, Q., Chin, M. L., Dubey, M. & Han, S. Black Phosphorus Radio-Frequency Transistors. *Nano Lett.* **14,** 6424–6429 (2014).
5. Das, S., Demarteau, M. & Roelofs, A. Ambipolar Phosphorene Field Effect Transistor. *ACS Nano* **8,** 11730–11738 (2014).
6. Zhu, W., Park, S., Yogeesh, M. N., McNicholas, K. M., Bank, S. R. & Akinwande, D. Black Phosphorus Flexible Thin Film Transistors at Gighertz Frequencies. *Nano Lett.* **16,** 2301–2306 (2016).
7. Ling, X., Liang, L., Huang, S., Puretzky, A. A., Geohegan, D. B., Sumpter, B. G., Kong, J., Meunier, V. & Dresselhaus, M. S. Low-Frequency Interlayer Breathing Modes in Few-

Layer Black Phosphorus. *Nano Lett.* **15,** 4080–4088 (2015).

8. Li, L. H., Cervenka, J., Watanabe, K., Taniguchi, T. & Chen, Y. Strong Oxidation Resistance of Atomically Thin Boron Nitride Nanosheets. *ACS Nano* **8,** 1457–1462 (2014).

9. Ahmed, F., Kim, Y. D., Choi, M. S., Liu, X., Qu, D., Yang, Z., Hu, J., Herman, I. P., Hone, J. & Yoo, W. J. High Electric Field Carrier Transport and Power Dissipation in Multilayer Black Phosphorus Field Effect Transistor with Dielectric Engineering. *Adv. Funct. Mater.* **27,** 1604025 (2017).

10. Liu, H., Neal, A. T., Zhu, Z., Luo, Z., Xu, X., Tománek, D. & Ye, P. D. Phosphorene: An Unexplored 2D Semiconductor with a High Hole Mobility. *ACS Nano* **8,** 4033–4041 (2014).

11. Kim, Y. D. *et al.* Bright visible light emission from graphene. *Nat. Nanotechnol.* **10,** 676–681 (2015).

12. Favron, A., Gaufrès, E., Fossard, F., Phaneuf-L'Heureux, A.-L., Tang, N. Y. W., Lévesque, P. L., Loiseau, A., Leonelli, R., Francoeur, S. & Martel, R. Photooxidation and quantum confinement effects in exfoliated black phosphorus. *Nat. Mater.* **14,** 826–832 (2015).
